# Impact of an artificial intelligence deep‐learning reconstruction algorithm for CT on image quality and potential dose reduction: A phantom study

**DOI:** 10.1002/mp.15807

**Published:** 2022-06-24

**Authors:** Joël Greffier, Salim Si‐Mohamed, Julien Frandon, Maeliss Loisy, Fabien de Oliveira, Jean Paul Beregi, Djamel Dabli

**Affiliations:** ^1^ IMAGINE UR UM 103, Montpellier University, Department of Medical Imaging Nîmes University Hospital Nîmes France; ^2^ University of Lyon INSA‐Lyon, University Claude Bernard Lyon 1, UJM‐Saint Etienne, CNRS, Inserm, CREATIS UMR 5220, U1206 Villeurbanne France; ^3^ Department of Radiology, Louis Pradel Hospital Hospices Civils de Lyon Bron France

**Keywords:** artificial Intelligence, deep‐learning image reconstruction algorithm, multidetector computed tomography, task‐based image quality assessment

## Abstract

**Background:**

Recently, computed tomography (CT) manufacturers have developed deep‐learning‐based reconstruction algorithms to compensate for the limitations of iterative reconstruction (IR) algorithms, such as image smoothing and the spatial resolution's dependence on contrast and dose levels.

**Purpose:**

To assess the impact of an artificial intelligence deep‐learning reconstruction (AI‐DLR) algorithm on image quality and dose reduction compared with a hybrid IR algorithm in chest CT for different clinical indications.

**Methods:**

Acquisitions on the CT American College of Radiology (ACR) 464 and CT Torso CTU‐41 phantoms were performed at five dose levels (CTDI_vol_: 9.5/7.5/6/2.5/0.4 mGy) used for chest CT conditions. Raw data were reconstructed using filtered backprojection, two levels of IR (iDose^4^ levels 4 (i4) and 7 (i7)), and five levels of AI‐DLR (Precise Image; Smoother, Smooth, Standard, Sharp, Sharper). Noise power spectrum (NPS), task‐based transfer function, and detectability index (*d*′) were computed: *d*′‐modeled detection of a soft tissue mediastinal nodule (low‐contrast soft tissue chest nodule within the mediastinum [LCN]), ground‐glass opacity (GGO), or high‐contrast pulmonary (HCP) lesion. The subjective image quality of chest anthropomorphic phantom images was independently evaluated by two radiologists. They assessed image noise, image smoothing, contrast between vessels and fat in the mediastinum for mediastinal images, visual border detection between bronchus and lung parenchyma for parenchymal images, and overall image quality using a commonly used four‐ or five‐point scale.

**Results:**

From Standard to Smoother levels, on average, the noise magnitude decreased (for all dose levels: −66.3% ± 0.5% for mediastinal images and −63.1% ± 0.1% for parenchymal images), the average NPS spatial frequency decreased (for all dose levels: −35.3% ± 2.2% for mediastinal images and −13.3% ± 2.2% for parenchymal images), and the detectability (*d*′) of the three lesions increased. The opposite pattern was found from Standard to Sharper levels. From Smoother to Sharper levels, the spatial resolution increased for the low‐contrast polyethylene insert and the opposite for the high‐contrast air insert. Compared to the i4 used in clinical practice, *d*′ values were higher using Smoother (mean for all dose levels: 338.7% ± 29.4%), Smooth (103.4% ± 11.2%), and Standard (34.1% ± 6.6%) levels for the LCN on mediastinal images and Smoother (169.5% ± 53.2% for GGO and 136.9% ± 1.6% for HCP) and Smooth (36.4% ± 22.1% and 24.1% ± 0.9%, respectively) levels for parenchymal images. Radiologists considered the images satisfactory for clinical use at these levels, but adaptation to the dose level of the protocol is required.

**Conclusion:**

With AI‐DLR, the smoothest levels reduced the noise and improved the detectability of chest lesions but increased the image smoothing. The opposite was found with the sharpest levels. The choice of level depends on the dose level and type of image: mediastinal or parenchymal.

**Abbreviations**
AI‐DLR: artificial intelligence deep‐learning reconstruction
CNN: convolutional neural network
CT: computed tomography
FBP: filtered backprojection
GGO: ground‐glass opacity
HCP: high‐contrast pulmonary
IR: iterative reconstruction
LCN: low‐contrast soft tissue chest nodule within the mediastinum
NPS: noise power spectrum
ROI: region of interest
SD: standard deviation
TTF: task‐based transfer function

## 1 INTRODUCTION

Recently, computed tomography (CT) manufacturers have developed deep‐learning‐based reconstruction (DLR) algorithms to compensate for the limitations of iterative reconstruction (IR) algorithms such as image smoothing and the spatial resolution's dependence on contrast and dose level.^1^, ^2^, ^3^ These DLR algorithms feature a deep neural network (DNN) to differentiate signal from image noise. In 2018, Canon Medical Systems developed the DLR–AiCE algorithm that trains DNNs with high‐quality model‐based IR images from patients.^1^ In 2019, GE Healthcare developed the TrueFidelity DLR algorithm that trains its DNN with high‐quality filtered backprojection (FBP) images from phantoms and patients.^2^

The first studies carried out on phantoms and patients with these two DLR algorithms have already demonstrated their contribution for improving image quality and their strong potential for dose reduction.^4^, ^5^, ^6^, ^7^, ^8^, ^9^, ^10^, ^11^, ^12^, ^13^, ^14^, ^15^, ^16^, ^17^, ^18^, ^19^, ^20^, ^21^, ^22^, ^23^, ^24^, ^25^, ^26^ Compared to IR algorithms, they reduce image noise whilst improving the contrast‐to‐noise ratio, which improves lesion detectability and diagnostic confidence.^5^, ^6^, ^19^, ^20^ In addition, phantom studies have also shown that image texture is preserved or improved and even approaches that obtained with FBP for TrueFidelity.^9^, ^11^, ^12^, ^13^, ^15^, ^23^, ^25^, ^27^ Other studies have also shown the strong potential of these algorithms in dose reduction, particularly with the implementation of low dose or ultra‐low dose protocols.^7^, ^21^, ^28^

Recently, Philips Healthcare also developed an artificial intelligence deep‐learning reconstruction (AI‐DLR) called Precise Image.^29^ This algorithm uses a convolutional neural network (CNN), which is a subtype of a DNN, where each layer performs convolution operation. For AI‐DLR, the CNN was trained to reproduce the image appearance (noise magnitude and noise texture) of routine‐dose FBP images from the raw data of low‐dose CT scans. For this, the CNN was trained with images at routine‐dose and low‐dose levels for the same patients. To avoid overexposing patients, low‐dose images were generated from the routine‐dose images using a simulation technique to accurately model photon and electronic noise.^29^ According to the manufacturer, the CNN was validated by comparing low‐dose images generated by AI‐DLR to routine‐dose images reconstructed using standard methods. To our knowledge, no studies have compared the impact of this AI‐DLR algorithm on dose reduction and image quality with an IR algorithm available for the same CT manufacturer.

The purpose of this study was to assess the impact on image quality and dose reduction potential of an AI‐DLR algorithm compared with a hybrid IR algorithm and the FBP. To do this, a task‐based image quality assessment was conducted in a geometric phantom and an anthropomorphic phantom (subjective image quality assessment) on chest CT conditions.

## 2 MATERIALS AND METHODS

### 2.1 Phantoms

**FIGURE 1:**
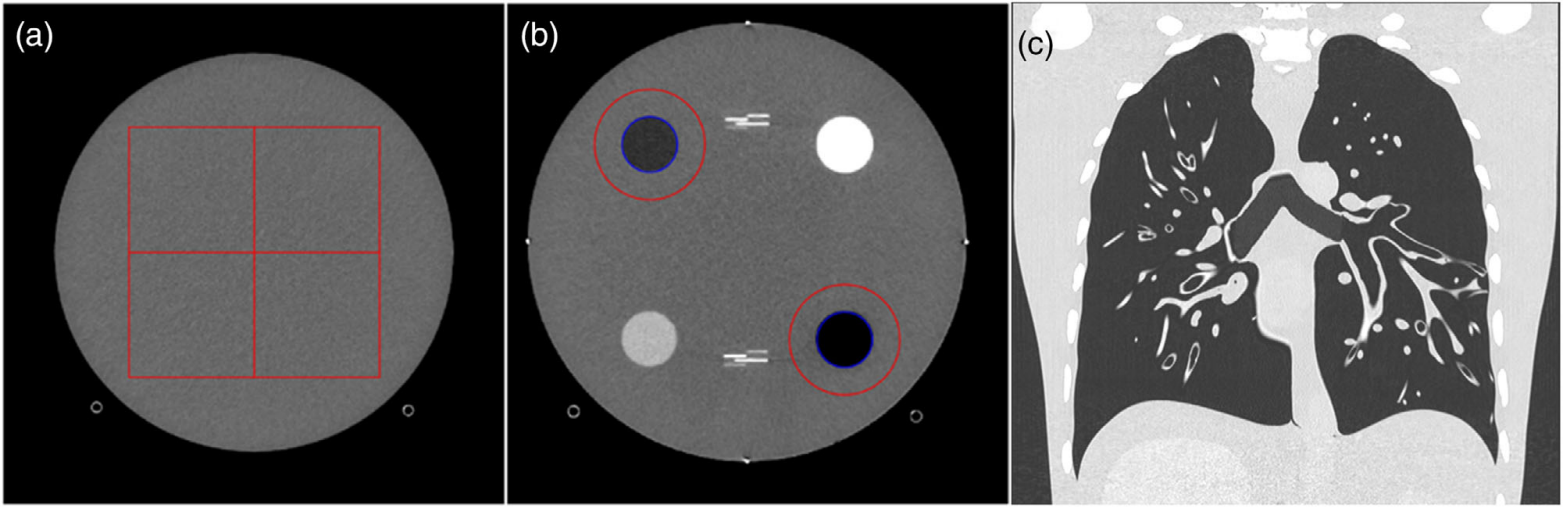
(a) Axial computed tomography (CT) image of the ACR phantom with regions of interest (ROIs) used for the noise power spectrum (NPS) assessment. (b) Axial CT image of the ACR phantom with ROIs used to calculate the task‐based transfer function (TTF) with air and polyethylene inserts. (c) Coronal chest CT image of the anthropomorphic phantom used for the subjective image quality assessment.

A 20‐cm diameter ACR QA phantom (Gammex 464) was used to perform a task‐based image quality assessment by measuring the noise power spectrum (NPS) (Figure 1a) and the task‐based transfer function (TTF) on air (−1000 HU) and polyethylene (−95 HU) inserts (Figure 1b). This phantom uses Solid Water, a durable water equivalent (±10 HU) for photon and electron energy measurements.

To evaluate the subjective image quality for the chest, an anthropomorphic phantom CT Torso CTU‐41 (Kyoto Kagaku; https://www.kyotokagaku.com/en/products_data/ph‐4/) was used (Figure 1c). The chest part was composed of different artificial organs such as the aorta, vena cava, trachea, heart, pulmonary vessels, and costal cartilage. Urethane‐based resin (SZ‐50) was used for the soft tissues and organs and epoxy‐based resin for synthetic bones. However, this phantom does not contain lung parenchyma and has a limited number of vessels and bronchi.

### 2.2 Acquisitions and reconstruction parameters

Images were acquired from both phantoms on an Incisive Premium CT system (Philips Healthcare) equipped with the fourth‐generation hybrid IR algorithm iDose (iDose^4^) and the Precise Image AI‐DLR.

All acquisitions were performed with a tube voltage of 120 kVp, a pitch factor of 1, beam collimation of 40 mm (64 × 0.625 mm), and rotation time of 0.35 s/rot. Tube current values (mA) were fixed and defined to obtain five values of volume CT dose indexes (CTDI_vol_: 9.5, 7.5, 6, 2.5, and 0.4 mGy). These are the dose levels used for chest CT examinations in clinical routine, for various clinical indications. The first three dose levels correspond to the 75th percentile, median, and 25th percentile of our national diagnostic reference levels for the chest CT. The last two dose levels correspond to the dose levels used in clinical routine for our low dose and ultra‐low dose chest CT protocols, respectively. For the ACR geometric phantom, five acquisitions were performed for each dose level and only one for the anthropomorphic phantom.

Raw data were reconstructed with FBP, the intermediate (i4; used in clinical routine) and highest (i7) levels of iDose^4^, and the five levels of AI‐DLR (Smoother, Smooth, Standard, Sharp, and Sharper). For the FBP and iDose^4^, the reconstruction kernel B usually used for mediastinal images and the reconstruction kernel YA usually used for parenchymal images were used. For AI‐DLR, the reconstruction kernels “soft tissue” and “lung” were used, respectively. With AI‐DLR, 1‐mm thick images can only be reconstructed with 50% overlap. For this purpose, the images of all algorithms used were reconstructed with a slice thickness of 1 mm (0.5‐mm overlap). Lastly, images were reconstructed using a field of view of 250 mm for the ACR phantom and 350 mm for the anthropomorphic phantom.

### 2.3 Task‐based image quality assessment—ACR geometric phantom

A task‐based image quality assessment was performed using the iQMetrix‐CT software developed by a working group from the French Society of Medical Physicists. No articles or reports have yet been published to describe the iQMetrix‐CT software; however, this software has been used in various studies.^11^, ^12^, ^30^

#### 2.3.1 Task‐based transfer function

In the iQMetrix‐CT software, the circular edge technique was used to calculate the TTF by plotting the Edge Spread Function (ESF).^31^ A conditioning of the ESF has been applied when the ESF was noisy. It consisted, by a simple mathematical operation, in making the ESF strictly monotonic. It was performed when the CNR_Total_, calculated on the composite image created from the average of the slices selected for the TTF calculation, was less than 15.^32^ Raw data ESF were then derived to obtain the line spread function (LSF). The Hann filter was applied to remove the noise on the tails of the LSF.^31^

In this study, the TTF was computed on the air and polyethylene inserts (Figure 1b) to get closer to the chest lesions.^33^, ^34^ To minimize the image‐noise effect on the ESF, the TTF was computed from 150 consecutive axial slices (30 slices for each of the 5 acquisitions). As the TTF was calculated from the images of the five acquisitions, no standard deviation (SD) or error bar could be calculated.

#### 2.3.2 Noise power spectrum

To use the same process than for the TTF calculation, the NPS was computed in 250 consecutive axial slices (50 slices for each of the 5 acquisitions) by placing 4 square regions of interest (ROIs) of 128 × 128 pixels (Figure 1a) using the same methodology as previously reported.^11^, ^12^, ^30^ In the iQMetrix‐CT software, the raw data NPS1D curves are fitted using an 11th‐order polynomial.

#### 2.3.3 Detectability index

A non‐prewhitening observer model with an eye filter (*d*′_NPWE_) was used to compute the detectability index of the chest lesions of 5‐mm diameter: low‐contrast soft tissue chest nodule within the mediastinum (LCN; 50 HU), ground‐glass opacity (GGO; 200 HU), and a high‐contrast pulmonary lesion (950 HU).^10^, ^12^ For the first two tasks, the TTF outcomes of the polyethylene insert (contrast with the Solid Water background material close to 95 HU) were used, whereas the TTF outcomes of the air insert (contrast with the Solid Water background material close to 1000 HU) were used for the third task. *d*′ of the LCN were computed only with the mediastinal images and the parenchymal images for the other two.

Interpretation conditions were defined in consensus by the radiologists of the study in reference to the visualization screen conditions used during the interpretation of chest CT images: a zoom factor of 1.5 and a 500‐mm viewing distance.

Other parameters used in the iQMetrix‐CT software to define each task function were a matrix size of 300 pixels and a pixel size of 0.05 mm, the “Designer” task function,^32^ and the Eckstein visual response function.^35^

#### 2.3.4 Relative differences between metrics

The mean relative differences (±SDs) between two values were computed for the five dose levels following the same methodology previously published.^36^ This allows comparison of the results obtained for each metric between two reconstruction algorithms for all dose levels.

### 2.4 Subjective image quality assessment on an anthropomorphic phantom

Two senior chest radiologists (12 and 8 years of experience) were read in consensus the chest images of the anthropomorphic phantom.^10^, ^12^ For each set of images, the radiologists read all the axial chest images randomly and were blinded to the reconstruction type (algorithm and levels) and dose level. They were instructed to subjectively assess image noise, image smoothing, and contrast between the vessels and fat in the mediastinum for mediastinal images and for visual border detection between bronchus and lung parenchyma for parenchymal images using a commonly used four‐ or five‐point scale.^10^ A value <3 was considered unsatisfactory for clinical use.

The radiologists first blindly read a set of images previously selected by the medical physicists from the geometric phantom results (highest and lowest dose; smoothest and least smooth image) to define how to score the images from the proposed scales. Sometime later, they performed the consensus reading.

## 3 RESULTS

NPS and TTF curves for all dose levels, all reconstruction types, and both reconstruction kernels are depicted in the Supplementary file. Images centered on Module 3 of the ACR phantom obtained for all types of reconstruction, for 0.4, 2.5, and 9.5 mGy and for both reconstruction kernels, are also depicted in the Supplementary file.

All values expressed as percentages hereafter correspond to the relative mean ± SD differences obtained for all dose levels between two algorithms or between two levels for the same algorithm.

### 3.1 Noise power spectrum

#### 3.1.1 Noise magnitude

**FIGURE 2:**
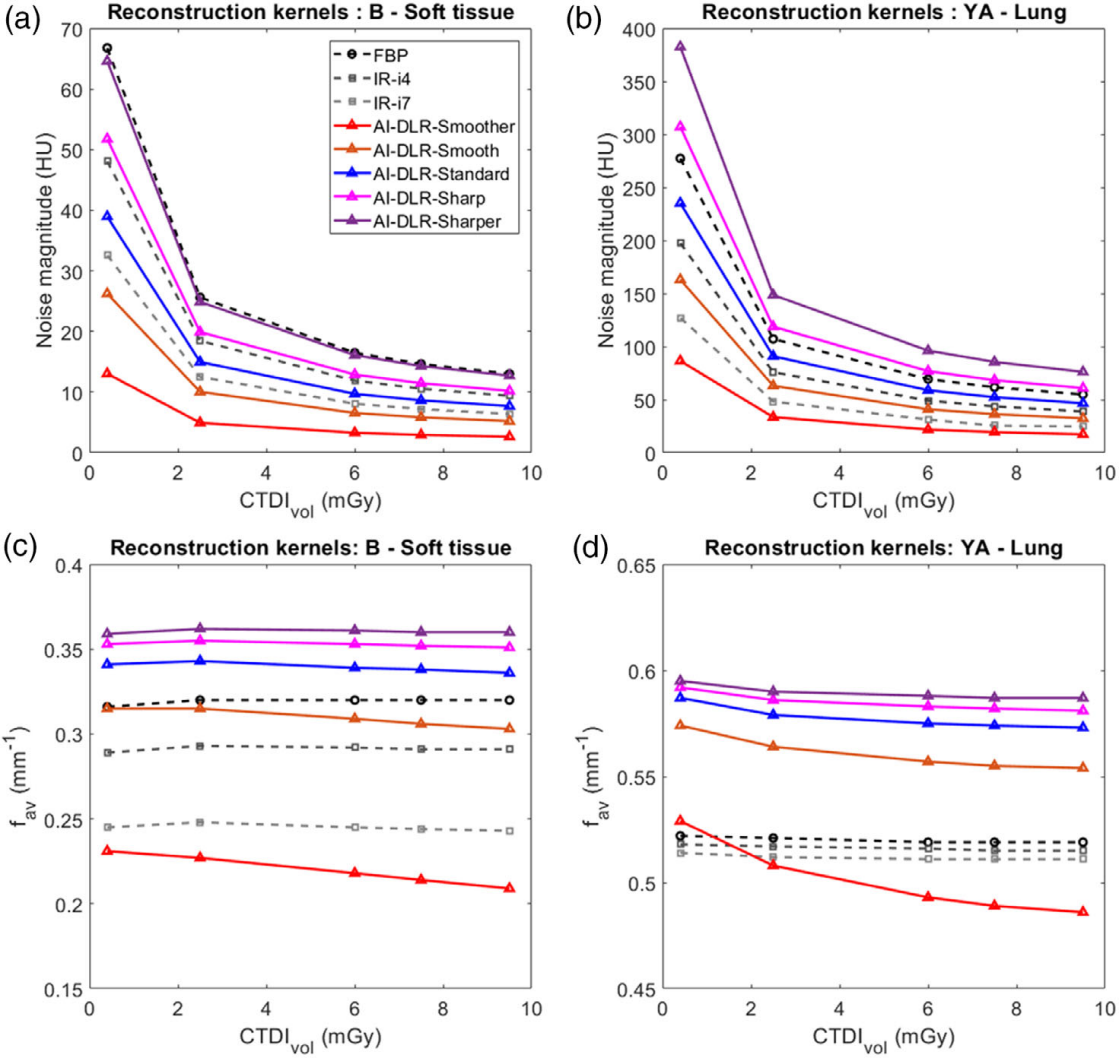
Noise magnitude (a and b) and average noise power spectrum spatial frequency (*f*_av_; c and d) obtained for all dose levels, all reconstruction types and both reconstruction kernels (Soft tissue kernels a and c and lung kernels b and d). i4 and i7 correspond to levels 4 and 7 of the iterative reconstruction (IR) algorithm iDose^4^; artificial intelligence deep‐learning reconstruction (AI‐DLR): Precise Image; noise texture.

For both reconstruction kernels and all reconstruction types, the noise magnitude decreased as the dose increased (Figure 2a,b).

For the soft tissue kernel and all dose levels (Figure 2a), the noise magnitude was lower than the FBP with i4 (−27.9% ± 0.1%) and i7 (−51.0% ± 0.2%), and similarly for the lung reconstruction kernel (Figure 2b), −29.2% ± 0.2% (i4) and −55.6% ± 1.8% (i7). For both kernels, the noise magnitude was lower with the Smoother level and increased from the Smoother to the Sharper level.

For the soft tissue kernel and all dose levels, the noise magnitude was lower with the Smoother (−59.6% ± 0.7%) and Smooth (−19.1% ± 0.5%) levels of AI‐DLR than i7. The noise magnitude of the Standard level was lower than i4 (−18.5% ± 0.4%) but higher than i7 (20.0% ± 0.3%). With the Sharp and Sharper levels, the noise magnitude was higher than i4 but lower than FBP.

For the lung kernel and all dose levels, the noise magnitude was lower with the Smoother level of AI‐DLR than i7 (−29.3% ± 3.1%). For the Smooth level, the noise magnitude was lower than i4 (−16.9% ± 0.3%) but higher than i7 (32.8% ± 5.6%). For the Standard level, the noise magnitude was lower than with FBP (−15.2% ± 0.1%) but higher than i4 (19.8% ± 0.4%). For the Sharp and Sharper levels, the noise magnitude was higher than with FBP: 11% ± 0% and 39% ± 0%, respectively.

For the soft tissue kernel and all dose levels (Figure 2c), the *f*_av_ values were lower with i4 (−8.8% ± 0.3%) and i7 (−23.2% ± 0.7%) than the FBP. For the lung reconstruction kernel (Figure 2d), similar *f*_av_ values were found with FBP, i4 (−0.7% ± 0.1%), and i7 (−1.6% ± 0.1%). For both kernels, *f*_av_ values were lower with the Smoother level of AI‐DLR, increased from the Smoother to the Sharper level, and decreased as the dose increased.

For the soft tissue kernel, *f*_av_ values obtained for the Smoother level of AI‐DLR were lower than those obtained with the FBP, i4, and i7, and for the Smooth level, they were only lower than the FBP.

For the lung kernel, *f*_av_ values were higher with all AI‐DLR levels than those obtained with the FBP, i4, and i7, except for the Smoother level from 2.5 to 9.5 mGy.

### 3.2 Task‐based transfer function

#### 3.2.1 Polyethylene insert

**FIGURE 3:**
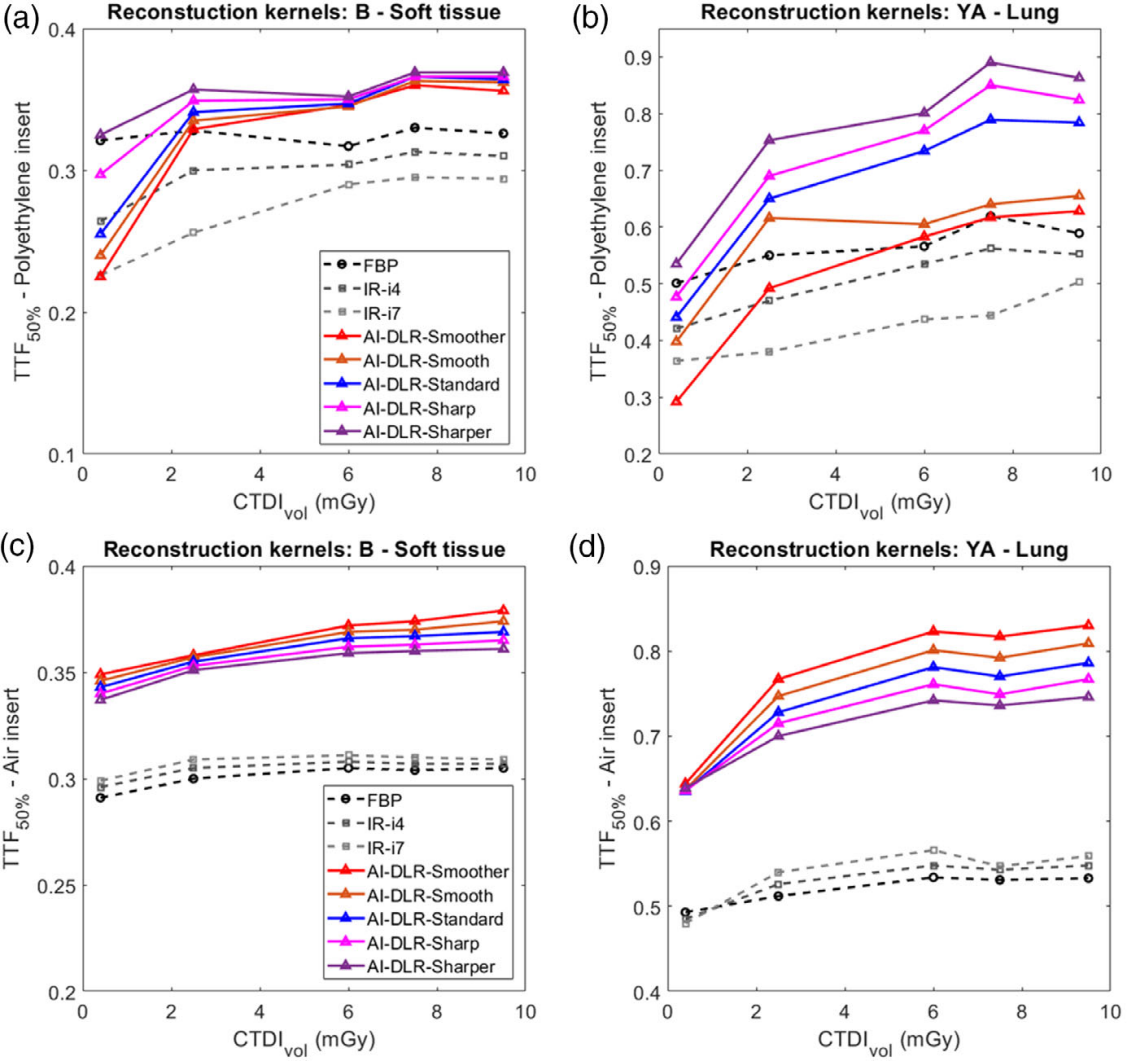
Values of task‐based transfer function at 50% (TTF_50%_) obtained for all dose levels, all reconstruction types and both reconstruction kernels: (a and b) Polyethylene insert for soft tissue and lung kernels, respectively; (c and d) air insert for soft tissue and lung kernels, respectively.

For both kernels and reconstruction types, the values of TTF_50%_ increased as the dose increased, especially for lung kernel (Figure 3a,b). Compared to FBP, TTF_50%_ values decreased as the iDose^4^ level increased.

For both kernels, the values of TTF_50%_ shifted toward higher frequencies from Smoother to Sharper, especially for the lung kernel.

For all dose levels, values of TTF_50%_ were higher with all AI‐DLR levels than with FBP, i4, and i7, except at 0.4 mGy for both reconstruction kernels. For the soft tissue kernel, values of TTF_50%_ at 0.4 mGy were higher than i4 only for the Sharp and Sharper levels for the Standard, Sharp, and Sharper levels for lung kernel.

#### 3.2.2 Air insert

For both kernels and all reconstruction types, the values of TTF_50%_ increased as the dose and as iDose^4^ level increased. For both kernels, values of TTF_50%_ were higher with iDose^4^ than with FBP and with AI‐DLR than with iDose^4^ and FBP (Figure 3c,d). Using AI‐DLR, values of TTF_50%_ shifted toward lower frequencies from Smoother to Sharper.

### 3.3 Detectability index

**FIGURE 4:**
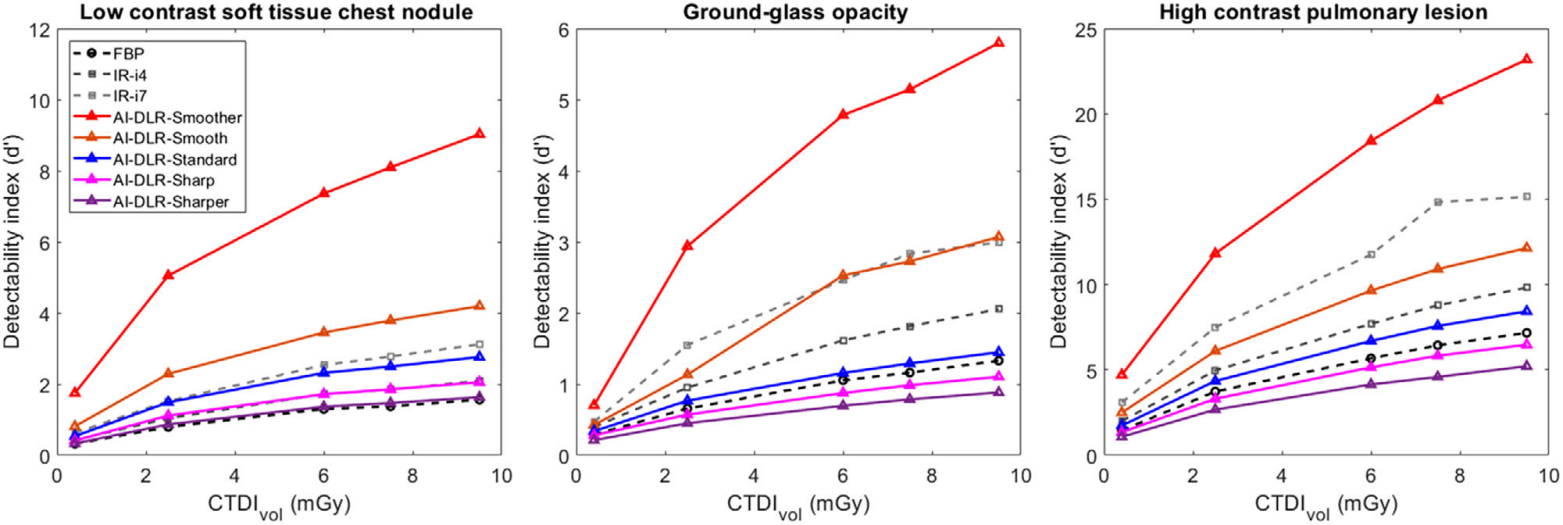
Detectability index (*d*′) values according to dose and reconstruction type for the detection of a low‐contrast soft tissue chest nodule within the mediastinum using soft tissue kernel (5 mm in diameter, −50‐HU contrast), the ground‐glass opacity (5 mm in diameter, −200‐HU contrast), and the high‐contrast pulmonary lesion (5‐mm diameter, −995‐HU contrast) using the lung kernel.

Regardless of the clinical task, the values of *d*′ increased as the dose and iDose^4^ level increased and from Sharper to Smoother (Figure 4). Compared to i4 usually used in clinical practice, *d*′ values obtained with AI‐DLR were higher with the Smoother and Smooth levels for all tasks. For the Standard level and all dose levels, *d*′ values were also higher than i4 for the LCN (34.1% ± 6.6%) and similar for the Sharp level (0.5% ± 4.1%).

Potentials to reduce the dose for all simulated chest lesions according to the AI‐DLR levels are depicted in Table 1.

**TABLE 1:** Potential dose reduction (%) with Smoother, Smooth, and Standard levels for the same *d*′ values obtained at 10 mGy with iDose^4^ level 4

	Smoother	Smooth	Standard
Low‐contrast soft tissue chest nodule within the mediastinum (%)	−94	−78	−57
Ground‐glass opacity (%)	−83	−58	–
High‐contrast pulmonary lesion (%)	−81	−46	–

Compared to *d*′ values obtained at 10 mGy and i4, similar *d*′ values were obtained at 0.62 mGy for the LCN, 1.67 mGy for the GGO, and 1.91 mGy for the HCN using the Smoother level. Using the Smooth level, similar *d*′ values were obtained at 2.22 mGy for the LCN, 4.15 mGy for the GGO, and 5.35 mGy for the HCN. For the Standard level, similar values were only obtained for *d*′ at 4.33 mGy for the LCN.

### 3.4 Subjective image quality assessment

Tables 2 and 3 show the outcomes of subjective image quality for mediastinal and parenchymal images of the anthropomorphic phantom.

**TABLE 2:** Results of subjective assessment of mediastinal images obtained by the two radiologists in consensus

			iDose^4^		Precise Image				
	CTDI_vol_ ^a^ (mGy)	FBP^b^	Level 4	Level 7	Smoother	Smooth	Standard	Sharp	Sharper
Image noise	0.4	**1**	**2**	**2.5**	4	3	**2.5**	**2**	**1**
	2.5	**2**	3	4	5	4.5	4	3	**2**
	6	**2.5**	3.5	4.5	5	5	4	4	3
	7.5	3.5	4	4.5	5	5	4.5	4	3.5
	9.5	3.5	3.5	4.5	5	5	4.5	4.5	3.5
Image smoothing	0.4	5	4.5	4	3	3.5	4.5	5	5
	2.5	5	4.5	3.5	**2**	3	4	4.5	4.5
	6	5	4.5	3.5	**2**	**2.5**	3.5	4	4.5
	7.5	5	4.5	3.5	**1.5**	**2.5**	3.5	4	4.5
	9.5	4.5	4	**2.5**	**1.5**	**2**	3	4	4
Contrast between the vessels and the fat in mediastinum	0.4	**1**	**1**	**2**	3.5	**2.5**	**1.5**	**1**	**1**
	2.5	**2**	**2.5**	3.5	4	3.5	3	**2.5**	**2**
	6	3	4	4	4.5	3.5	4	3.5	3
	7.5	3.5	4.5	5	4.5	4	4	4	3.5
	9.5	4	4.5	4.5	3.5	4	4.5	4.5	3.5
Overall image quality	0.4	**1**	**1**	**2**	3	**2.5**	**1.5**	**1.5**	**1**
	2.5	**2**	**2.5**	3	**2.5**	3	3	**2.5**	**2**
	6	**2.5**	3.5	4	**2.5**	3	3	3.5	3
	7.5	3.5	4.5	4.5	**1.5**	3	3.5	3.5	3.5
	9.5	3.5	4	3.5	**1**	**2**	3.5	4.5	3.5

*Note*: Bold indicates values <3, which were considered unsatisfactory for clinical use.

^a^ Volume CT dose index.

^b^ Filtered back projection.

**TABLE 3:** Results of subjective assessment of parenchymal images obtained by the two radiologists in consensus

			iDose^4^		Precise Image				
	CTDI_vol_ ^a^ (mGy)	FBP^b^	Level 4	Level 7	Smoother	Smooth	Standard	Sharp	Sharper
Image noise	0.4	**1.5**	**2**	3	5	4	3.5	3	**2**
	2.5	3	4	4.5	5	5	5	4	3.5
	6	4	4.5	4.5	5	5	5	4.5	4
	7.5	4	4.5	5	5	5	5	4.5	4
	9.5	5	5	5	5	5	5	5	5
Image smoothing	0.4	5	4.5	4	4	4.5	4.5	5	5
	2.5	5	4.5	4	**2.5**	4	4	4.5	4.5
	6	4.5	4.5	3.5	**2.5**	3.5	4	4.5	4.5
	7.5	5	4.5	3.5	**2.5**	3.5	4	5	5
	9.5	5	4.5	**2.5**	**2**	3	3.5	4.5	5
Visual border detection between bronchus and lung parenchyma	0.4	**2**	**2**	**2**	4	4.5	3.5	**2.5**	**2**
	2.5	3.5	4	3	4	5	5	4.5	4.5
	6	4	4.5	4	4.5	5	5	5	4.5
	7.5	4.5	4	4	4.5	5	5	5	5
	9.5	4.5	4	4	4.5	5	5	5	5
Overall image quality	0.4	**2**	**2.5**	**2.5**	4	4.5	3.5	3	**2.5**
	2.5	3	3.5	3.5	3.5	4.5	4.5	4	4
	6	3.5	4.5	4.5	3.5	4.5	4.5	4.5	4
	7.5	4	4	4	3	3	4.5	4.5	4.5
	9.5	4	4	3.5	**2.5**	3	3.5	4	4.5

*Note*: Bold indicates values <3, which were considered unsatisfactory for clinical use.

^a^ Volume CT dose index.

^b^ Filtered back projection.

**FIGURE 5:**
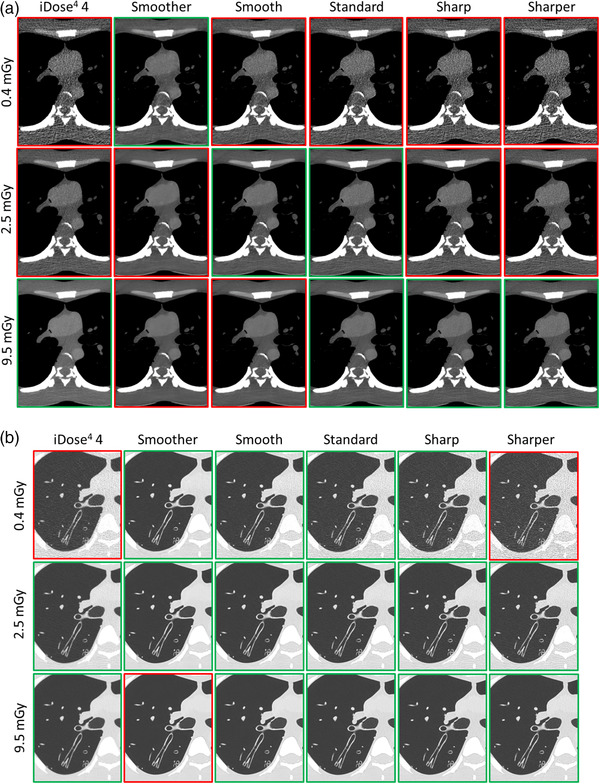
Anthropomorphic phantom CT images in the axial plane obtained for the level 4 of iDose^4^ and the five levels of Precise Image and at 0.4, 2.5, and 9.5 mGy: (a) Mediastinal images (WL: 370 HU, WW: 60 HU) were centered on the anterior vascular mediastinum structures; (b) parenchymal images (WL: −1600 HU, WW: 600 HU) were centered at the level of the tracheal carina. Red contours correspond to images with an overall image quality score considered unsatisfactory for clinical use and green contours the opposite.

For the mediastinal images using AI‐DLR (Table 1 and Figure 5a), the image noise decreased as the dose increased, and the opposite for image smoothing and contrast. Image smoothing was rated lower than 3 for all dose levels with the Smoother level (except at 0.4 mGy) and from 6 to 9.5 mGy for the Smooth level. At 0.4 mGy, the overall image quality was rated higher or equal to 3 only for the Smoother level. For this level, the score was rated lower than 3 for other dose levels regarding the image smoothing score. Regarding the contrast score, it was rated lower than 3 at 0.4 and 2.5 mGy for the Sharp and Sharper levels and at 0.4 mGy for the Standard level.

Based on overall image quality, the mediastinum was best viewed at the lowest dose (0.4 mGy) with the Smoother level. Intermediated dose levels (2.5–7 mGy) were best viewed at smooth or standard levels and the highest dose levels (6–9.5) were best viewed with Standard, Sharp, or Sharper levels. An improvement with AI‐DLR was noted at 0.4 and 2.5 mGy compared to i4 and i7, which demonstrated unacceptable image quality at these doses.

For parenchymal images using AI‐DLR (Table 2 and Figure 5b), image noise decreased as the dose increased and the opposite for visual detection of the border between bronchial and lung parenchyma. Image smoothing was rated above average or excellent for all dose levels using FBP, i4, and the Sharp and Sharper levels of AI‐DLR. For the other AI‐DLR levels and i7, image smoothing increased as the dose increased. Compared to i4 and i7, the use of AI‐DLR improved the visual border detection between bronchus and lung parenchyma. Overall image quality was rated 3 or above for all AI‐DLR levels apart from 9 mGy with the Smoother level and 0.4 mGy for the Sharper level. Based on the image smoothing outcomes, the overall image quality decreased as the dose increased for the Smoother and Smooth levels of AI‐DLR.

Finally, all levels of AI‐DLR can be used for low‐dose protocols and all levels for chest ultra‐low dose protocol, except the Sharper level. For the ultralow dose, the overall image quality was not considered clinically acceptable with i4 and i7.

## 4 DISCUSSION

In the present study, we assessed the impact of a new AI‐DLR algorithm on image quality, in comparison to a standard clinical protocol using either FBP or IR reconstruction algorithms in a phantom study. Task‐based and subjective image quality assessments were performed in chest CT conditions for the evaluation of different clinical tasks. We evaluated the five levels available and characterized for each of them a different impact on noise magnitude, noise texture, and spatial resolution according to the contrast and detectability of simulated lung lesions. From Standard to Smoother levels, the noise decreased and detectability increased but the image texture was modified and smoothed, and the opposite for the Standard and Sharper levels. Compared to the usual routine iterative algorithm, the Smoother, Smooth, and Standard levels produced better image quality and had a greater dose reduction potential for the optimization of routine CT protocols.

The NPS results showed that the amplitude and texture of the noise varied with the level of AI‐DLR. The variations of noise magnitude and *f*_av_ values obtained with AI‐DLR may have been related to the difference in non‐stationarity of the noise between their levels, especially with the Smoother level. The names given to the various levels of the AI‐DLR by the manufacturer clearly reflect the impact of these levels on the images and were close to the behavior of the reconstruction kernels. In one direction, the image was less noisy but smoother with few details and, in the other direction, the image was noisier but with sharper definition and finer details. These results were confirmed by both chest radiologists during the subjective assessment of the image noise and the image smoothing on the images of the anthropomorphic phantom. The impact of the change in noise was also greater on mediastinal images than on parenchymal images. For the mediastinal images, radiologists even considered the low‐dose images (0.4 and 2.5 mGy) with the Sharp and Sharper levels as unsatisfactory for clinical use due to too much noise in the image, and the high‐dose images (9.5 mGy) with Smoother level were considered unsatisfactory due to too much image smoothing and the appearance of distortion particularly in the interface between soft tissue and air. For parenchymal images, only images reconstructed with the Smoother level were considered too smooth and with artifacts from 2.5 to 9.5 mGy. However, at 0.4 mGy, the image smoothing was rated above average for the Smoother level. This behavior was also found for other levels of AI‐DLR where image smoothing was rated less important as the dose decreased, and therefore the noise increased. Furthermore, compared to iDose^4^ and, in particular, the level 4 used in clinical routine, we found that the noise magnitude was lower only at the Smoother and Smooth levels for the two reconstruction kernels and the Standard level only for the soft tissue kernel. On the other hand, the *f*_av_ was higher than iDose^4^ level 4 for all levels except for the Smoother level. Lastly, the noise variations between this algorithm and the iterative algorithm usually used in clinical routine were different from those found in the literature, with the other two DLR algorithms.^9^, ^11^, ^12^, ^13^, ^15^, ^23^, ^25^ Indeed, with TrueFidelity and AiCE, the noise was lower compared to ASIR‐V 50% and AIDR‐3D Enhanced, respectively, and this was more so as the strength level of each algorithm increased. With TrueFidelity, the *f*_av_ values were higher than ASIR‐V 50% and the difference increased with the level. For AiCE, the *f*_av_ values were higher than for AIDR‐3D Enhanced but only for the Mild and Standard levels.^11^

The TTF results showed that, as with iDose^4^, AI‐DLR has nonlinear properties despite the fact that it results in CNN with FBP images. For both algorithms, the spatial resolution depended on the contrast of the insert and the level of dose and noise. Indeed, for both inserts studied, the TTF_50%_ values increased as the dose increased, and therefore the noise decreased. This variation was more marked for the low‐contrast insert than for the high‐contrast insert and for the kernel lung than for the soft tissue kernel. These results are directly related to the circular edge technique used to calculate the TTF by plotting the ESF.^31^ Both the ESF and the TTF are influenced by the amount of image noise and even more so when the difference in contrast between the insert and the background is low.^32^, ^37^ Moreover, the opposite behavior is found depending on the insert used and according to the AI‐DLR level. With the low‐contrast insert, the TTF was higher with the Sharper level and decreased as Smoother was approached. The opposite was found for the high‐contrast insert. However, the variation in TTF_50%_ values with level was less pronounced for all reconstruction kernels. The impact of AI‐DLR level on TTF_50%_ values was also more pronounced with the low‐contrast insert and the lung kernel. These results were not directly found by the radiologists with the subjective image quality assessment. For mediastinal images, better contrast between fat and vessels was found with Standard, Smooth, and Smoother levels than with Sharp and Sharper. For parenchymal images, radiologists found visual border detection between bronchus and lung parenchyma above average or excellent at all dose and AI‐DLR levels, except at 0.4 mGy with Standard, Sharp, and Sharper levels. Finally, compared to iDose^4^ level 4 used in clinical routine, TTF_50%_ values were higher with AI‐DLR for both kernels and both inserts, except at 0.4 mGy for Standard, Smooth, and Smoother levels. These variations in values between AI‐DLR and iDose^4^ were similar to those found between TrueFidelity and ASIR‐V 50% for two low and high contrast inserts in a homemade phantom.^23^ In addition, the large variations according to the level were close to those found with AiCE.

The detectability index results confirmed the variations in NPS and TTF with AI‐DLR levels. The differences between the AI‐DLR levels were directly related to the variations in noise magnitude, more marked between Smoother and Smooth levels and Smooth and Standard levels. Compared to i4 used in clinical routine, the *d*′ values were higher with Smoother, Smooth, and Standard levels and equivalent with Sharp level for mediastinal images. For parenchymal images, *d*′ values were higher than i4 only for Smoother and Smooth levels. The highest *d*′ values were found for the same lesions simulated with TrueFidelity and AiCE^12^ compared to IR algorithms. These differences may be explained by the different software used and the parameters selected to define each task function including the matrix and pixel size and the type of task function. Matrix size and pixel size have a significant impact on *d*′ values. However, a change in these parameters does not affect the variation in *d*′ values according to the dose level or the type or level of algorithm.

These results for *d*′ could be linked to the results of the overall image quality assessment performed by the two radiologists to choose which level to use in clinical practice. For mediastinal images, the image quality was rated satisfactory for clinical use with the Standard and Sharp levels for dose levels from 6 to 9.5 mGy, regularly used in clinical practice. In addition to image smoothing, they reported image distortion and nonlinearity of the beam hardening correction with the Smooth and Smoother levels at the highest dose levels. Using these levels should therefore be limited from 7.5 to 2.5 mGy for Smooth and 0.4 mGy for Smoother levels, respectively. For parenchymal images, the image quality was also rated as satisfactory for clinical use with both Smooth and Smoother levels for all dose levels except for 9.5 mGy with Smoother. They reported that image smoothing was more pronounced as the noise decreased with increasing dose. The Smooth level can therefore be used in clinical routine for all dose levels, and the Smoother level with a smoother and less noisy image. The results obtained in the present study with the ACR phantom and the anthropomorphic phantom should be taken with caution. Both phantoms did not perfectly mimic the patient's body morphology and anatomical structures. The inserts used with the ACR phantom to simulate lesions did not precisely mimic the patients’ anatomical structures. The contrast of the simulated chest lesions was slightly different from the contrast between each insert and the background material of the phantom used for the TTF calculation. However, this small difference in contrast has a limited impact on the calculation of *d*′, which is strongly influenced by the NPS results. The anthropomorphic phantom has a low body mass index (18 kg/m^2^), no lung parenchyma or fat, and no real lesions. Patient studies should now be carried out to confirm these phantom results and validate the choice of level for our three routine chest CT protocols.

This study has its limitations. The image quality and the anthropomorphic phantoms used are very useful for image quality assessment; however, their size and water‐equivalent diameter (WED) (mean WED of 20.1 ± 0.3 cm and 21.8 ± 1.7 cm, respectively) are far from those of patients undergoing a chest CT examination^38^ (WED close to 26 cm). The five dose levels were defined without taking the WED differences into account, which could lead to an overestimation of the results obtained. In addition, to ensure a fixed CTDI_vol_, we had set the mAs, and the tube current modulation was not used. Different results may have been found for phantoms of different sizes and if the modulation system had been activated. We therefore chose to calculate the detectability index in the frequency domain using the NPWE model observer as recommended by the AAPM.^32^ However, to get as close as possible to patient images, it would have been more realistic to calculate the index via a model observer in image space from an anthropomorphic phantom with real lesions. To evaluate the image quality obtained with these new reconstruction algorithms in a realistic way, another way would be to use textured phantoms with more realistic tissue texture and anatomical features.^39^, ^40^, ^41^ However, these phantoms were not available in our institution. Last, the assumptions of non‐stationarity of noise for the different algorithms used in this study were not studied and could be the subject of a future study.^25^

## 5 CONCLUSION

In the present study, we evaluated the impact on image quality of an AI‐DLR algorithm. The choice of level of this algorithm impacted in different ways the noise magnitude and noise texture, the spatial resolution, and the detectability of simulated lung lesions. Compared to the IR algorithm used in clinical practice, the detectability of simulated lung lesions was better with the smoothest levels, confirming an improvement in image quality for the same dose level. Potentials for dose reduction were found with the Smoother (from −81% to −94%) and Smooth (from −46% to −78%) levels for all simulated lung lesions. Patient studies are now required to confirm the choice of AI‐DLR levels defined in this study in relation to dose level.

## CONFLICT OF INTEREST

The authors declare that there is no conflict of interest that could be perceived as prejudicing the impartiality of the research reported.

## FUNDING INFORMATION

The authors state that this work has not received any funding.

## Supporting information




**Figure 1‐SM**. Noise power spectrum (NPS) curves obtained for all dose levels, all reconstruction types and both reconstruction kernels
**Figure 2‐SM**. Task‐based transfer function (TTF) curves of the polyethylene and the air inserts obtained for all dose levels, all reconstruction types and both reconstruction kernels

## Data Availability

Authors will share data upon request to the corresponding author.

## References

[1] Boedeker K . AiCE deep learning reconstruction: bringing the power of ultra‐high resolution CT to routine imaging. In: *Technical White Paper on Deep Learning Reconstruction Canon Medical System*. 2019.

[2] Hsieh J , Liu E , Nett B , Tang J , Thibault JB , Sahney S . A new era of image reconstruction: TrueFidelity™. In: *Technical White Paper on Deep Learning Image Reconstruction*. 2019.

[3] Willemink MJ , Noel PB . The evolution of image reconstruction for CT‐from filtered back projection to artificial intelligence. Eur Radiol. 2019;29(5):2185‐2195. [published online ahead of print 2018/11/01]. 30377791 10.1007/s00330-018-5810-7 PMC6443602

[4] Akagi M , Nakamura Y , Higaki T , Narita K , Honda Y , Awai K . Deep learning reconstruction of equilibrium phase CT images in obese patients. Eur J Radiol. 2020;133:109349. [published online ahead of print 2020/11/06]. 33152626 10.1016/j.ejrad.2020.109349

[5] Akagi M , Nakamura Y , Higaki T , et al. Deep learning reconstruction improves image quality of abdominal ultra‐high‐resolution CT. Eur Radiol. 2019;29(11):6163‐6171. [published online ahead of print 2019/04/13]. 30976831 10.1007/s00330-019-06170-3

[6] Benz DC , Benetos G , Rampidis G , et al. Validation of deep‐learning image reconstruction for coronary computed tomography angiography: impact on noise, image quality and diagnostic accuracy. J Cardiovasc Comput Tomogr. 2020;14(5):444‐451. [published online ahead of print 2020/01/25]. 31974008 10.1016/j.jcct.2020.01.002

[7] Bernard A , Comby PO , Lemogne B , et al. Deep learning reconstruction versus iterative reconstruction for cardiac CT angiography in a stroke imaging protocol: reduced radiation dose and improved image quality. Quant Imaging Med Surg. 2021;11(1):392‐401. [published online ahead of print 2021/01/05]. 33392038 10.21037/qims-20-626 PMC7719916

[8] Brady SL , Trout AT , Somasundaram E , Anton CG , Li Y , Dillman JR . Improving image quality and reducing radiation dose for pediatric CT by using deep learning reconstruction. Radiology. 2021;298(1):180‐188. [published online ahead of print 2020/11/18]. 33201790 10.1148/radiol.2020202317

[9] Franck C , Zhang G , Deak P , Zanca F . Preserving image texture while reducing radiation dose with a deep learning image reconstruction algorithm in chest CT: a phantom study. Phys Med. 2021;81:86‐93. [published online ahead of print 2021/01/15]. 33445125 10.1016/j.ejmp.2020.12.005

[10] Greffier J , Boccalini S , Beregi JP , et al. CT dose optimization for the detection of pulmonary arteriovenous malformation (PAVM): a phantom study. Diagn Interv Imaging. 2020;101(5):289‐297. [published online ahead of print 2020/01/15]. 31932228 10.1016/j.diii.2019.12.009

[11] Greffier J , Dabli D , Frandon J , et al. Comparison of two versions of a deep learning image reconstruction algorithm on CT image quality and dose reduction: a phantom study. Med Phys. 2021;48(10):5743‐5755. [published online ahead of print 2021/08/22]. 34418110 10.1002/mp.15180

[12] Greffier J , Frandon J , Si‐Mohamed S , et al. Comparison of two deep learning image reconstruction algorithms in chest CT images: a task‐based image quality assessment on phantom data. Diagn Interv Imaging. 2022;103:21‐30. 10.1016/j.diii.2021.08.001. [published online ahead of print 2021/09/09]. 34493475

[13] Greffier J , Hamard A , Pereira F , et al. Image quality and dose reduction opportunity of deep learning image reconstruction algorithm for CT: a phantom study. Eur Radiol. 2020;30(7):3951‐3959. [published online ahead of print 2020/02/27]. 32100091 10.1007/s00330-020-06724-w

[14] Hata A , Yanagawa M , Yoshida Y , et al. The image quality of deep‐learning image reconstruction of chest CT images on a mediastinal window setting. Clin Radiol. 2021;76(2):155.e115‐155.e123. [published online ahead of print 2020/11/23]. 10.1016/j.crad.2020.10.011 33220941

[15] Higaki T , Nakamura Y , Zhou J , et al. Deep learning reconstruction at CT: phantom study of the image characteristics. Acad Radiol. 2020;27(1):82‐87. [published online ahead of print 2019/12/11]. 31818389 10.1016/j.acra.2019.09.008

[16] Ichikawa Y , Kanii Y , Yamazaki A , et al. Deep learning image reconstruction for improvement of image quality of abdominal computed tomography: comparison with hybrid iterative reconstruction. Jpn J Radiol. 2021;39:598‐604. 10.1007/s11604-021-01089-6. [published online ahead of print 2021/01/16]. 33449305

[17] Jensen CT , Liu X , Tamm EP , et al. Image quality assessment of abdominal CT by use of new deep learning image reconstruction: initial experience. AJR Am J Roentgenol. 2020;215(1):50‐57. [published online ahead of print 2020/04/15]. 32286872 10.2214/AJR.19.22332

[18] Kawashima H , Ichikawa K , Takata T , et al. Performance of clinically available deep learning image reconstruction in computed tomography: a phantom study. J Med Imaging (Bellingham). 2020;7(6):063503. [published online ahead of print 2020/12/22]. 33344672 10.1117/1.JMI.7.6.063503 PMC7739999

[19] Kim I , Kang H , Yoon HJ , Chung BM , Shin NY . Deep learning‐based image reconstruction for brain CT: improved image quality compared with adaptive statistical iterative reconstruction‐Veo (ASIR‐V). Neuroradiology. 2021;63:905‐912. 10.1007/s00234-020-02574-x. [published online ahead of print 2020/10/11]. 33037503

[20] Kim JH , Yoon HJ , Lee E , Kim I , Cha YK , Bak SH . Validation of deep‐learning image reconstruction for low‐dose chest computed tomography scan: emphasis on image quality and noise. Korean J Radiol. 2021;22(1):131‐138. [published online ahead of print 2020/07/31]. 32729277 10.3348/kjr.2020.0116 PMC7772377

[21] Lenfant M , Chevallier O , Comby PO , et al. Deep learning versus iterative reconstruction for CT pulmonary angiography in the emergency setting: improved image quality and reduced radiation dose. Diagnostics (Basel). 2020;10(8):558. [published online ahead of print 2020/08/08]. 10.3390/diagnostics10080558 PMC7460033 32759874

[22] Nakamura Y , Higaki T , Tatsugami F , et al. Deep learning–based CT image reconstruction: initial evaluation targeting hypovascular hepatic metastases. Radiol Artif Intell. 2019;1(6):e180011. 33937803 10.1148/ryai.2019180011 PMC8017421

[23] Racine D , Becce F , Viry A , et al. Task‐based characterization of a deep learning image reconstruction and comparison with filtered back‐projection and a partial model‐based iterative reconstruction in abdominal CT: a phantom study. Phys Med. 2020;76:28‐37. [published online ahead of print 2020/06/24]. 32574999 10.1016/j.ejmp.2020.06.004

[24] Singh R , Digumarthy SR , Muse VV , et al. Image quality and lesion detection on deep learning reconstruction and iterative reconstruction of submillisievert chest and abdominal CT. AJR Am J Roentgenol. 2020;214(3):566‐573. [published online ahead of print 2020/01/23]. 31967501 10.2214/AJR.19.21809

[25] Solomon J , Lyu P , Marin D , Samei E . Noise and spatial resolution properties of a commercially available deep learning‐based CT reconstruction algorithm. Med Phys. 2020;47:3961‐3971. 10.1002/mp.14319. [published online ahead of print 2020/06/09]. 32506661

[26] Tatsugami F , Higaki T , Nakamura Y , et al. Deep learning‐based image restoration algorithm for coronary CT angiography. Eur Radiol. 2019;29(10):5322‐5329. [published online ahead of print 2019/04/10]. 30963270 10.1007/s00330-019-06183-y

[27] Noda Y , Kaga T , Kawai N , et al. Low‐dose whole‐body CT using deep learning image reconstruction: image quality and lesion detection. Br J Radiol. 2021;94(1121):20201329. [published online ahead of print 2021/02/12]. 33571010 10.1259/bjr.20201329 PMC8506192

[28] Cao L , Liu X , Li J , et al. A study of using a deep learning image reconstruction to improve the image quality of extremely low‐dose contrast‐enhanced abdominal CT for patients with hepatic lesions. Br J Radiol. 2021;94(1118):20201086. [published online ahead of print 2020/11/27]. 33242256 10.1259/bjr.20201086 PMC7934287

[29] White paper – AI for significantly lower dose and improvement image quality – Precise Image. *Philips – Computed Tomography*; 2021. https://www.philips.com/cdam/b2bhc/master/resource-catalog/landing/precise-suite/incisive_precise_image.pdf

[30] Greffier J , Frandon J , Sadate A , et al. Impact of four kVp combinations available in a dual‐source CT on the spectral performance of abdominal imaging: a task‐based image quality assessment on phantom data. J Appl Clin Med Phys. 2021;22(8):243‐254. [published online ahead of print 2021/07/28]. 34312979 10.1002/acm2.13369 PMC8364263

[31] Richard S , Husarik DB , Yadava G , Murphy SN , Samei E . Towards task‐based assessment of CT performance: system and object MTF across different reconstruction algorithms. Med Phys. 2012;39(7):4115‐4122. [published online ahead of print 2012/07/27]. 22830744 10.1118/1.4725171

[32] Samei E , Bakalyar D , Boedeker KL , et al. Performance evaluation of computed tomography systems: summary of AAPM Task Group 233. Med Phys. 2019;46(11):e735‐e756. [published online ahead of print 2019/08/14]. 31408540 10.1002/mp.13763

[33] Samei E , Richard S . Assessment of the dose reduction potential of a model‐based iterative reconstruction algorithm using a task‐based performance metrology. Med Phys. 2015;42(1):314‐323. [published online ahead of print 2015/01/08]. 25563271 10.1118/1.4903899

[34] Verdun FR , Racine D , Ott JG , et al. Image quality in CT: from physical measurements to model observers. Phys Med. 2015;31(8):823‐843. [published online ahead of print 2015/10/16]. 26459319 10.1016/j.ejmp.2015.08.007

[35] Eckstein M , Bartroff J , Abbey C , Whiting J , Bochud F . Automated computer evaluation and optimization of image compression of x‐ray coronary angiograms for signal known exactly detection tasks. Opt Express. 2003;11(5):460‐475. [published online ahead of print 2003/03/10]. 19461753 10.1364/oe.11.000460

[36] Greffier J , Viry A , Barbotteau Y , et al. Phantom task‐based image quality assessment of three generations of rapid kV‐switching dual‐energy CT systems on virtual monoenergetic images. Med Phys. 2022;49(4):2233‐2244. [published online ahead of print 2022/02/21]. 35184293 10.1002/mp.15558

[37] Maidment AD , Albert M . Conditioning data for calculation of the modulation transfer function. Med Phys. 2003;30(2):248‐253. [published online ahead of print 2003/02/28]. 12607842 10.1118/1.1534111

[38] Si‐Mohamed S , Dupuis N , Tatard‐Leitman V , et al. Virtual versus true non‐contrast dual‐energy CT imaging for the diagnosis of aortic intramural hematoma. Eur Radiol. 2019;29(12):6762‐6771. [published online ahead of print 2019/07/03]. 31264015 10.1007/s00330-019-06322-5

[39] Solomon J , Ba A , Bochud F , Samei E . Comparison of low‐contrast detectability between two CT reconstruction algorithms using voxel‐based 3D printed textured phantoms. Med Phys. 2016;43(12):6497. [published online ahead of print 2016/12/03]. 27908164 10.1118/1.4967478

[40] Mei K , Geagan M , Roshkovan L , et al. Three‐dimensional printing of patient‐specific lung phantoms for CT imaging: emulating lung tissue with accurate attenuation profiles and textures. Med Phys. 2022;49(2):825‐835. [published online ahead of print 2021/12/16]. 34910309 10.1002/mp.15407 PMC8828694

[41] Hernandez‐Giron I , den Harder JM , Streekstra GJ , Geleijns J , Veldkamp WJH . Development of a 3D printed anthropomorphic lung phantom for image quality assessment in CT. Phys Med. 2019;57:47‐57. [published online ahead of print 2019/02/11]. 30738531 10.1016/j.ejmp.2018.11.015

