# Supplementary material for: Impact of an artificial intelligence deep‐learning reconstruction algorithm for CT on image quality and potential dose reduction: A phantom study
Source: Med Phys. 2022 Jun 24;49(8):5052–63. doi: 10.1002/mp.15807 (PMC9544990; doi:10.1002/mp.15807)

**Supplementary Material**

**Figure 1-SM.** Noise power spectrum (NPS) curves obtained for all dose levels, all reconstruction types and both reconstruction kernels

- *Soft tissue kernel*


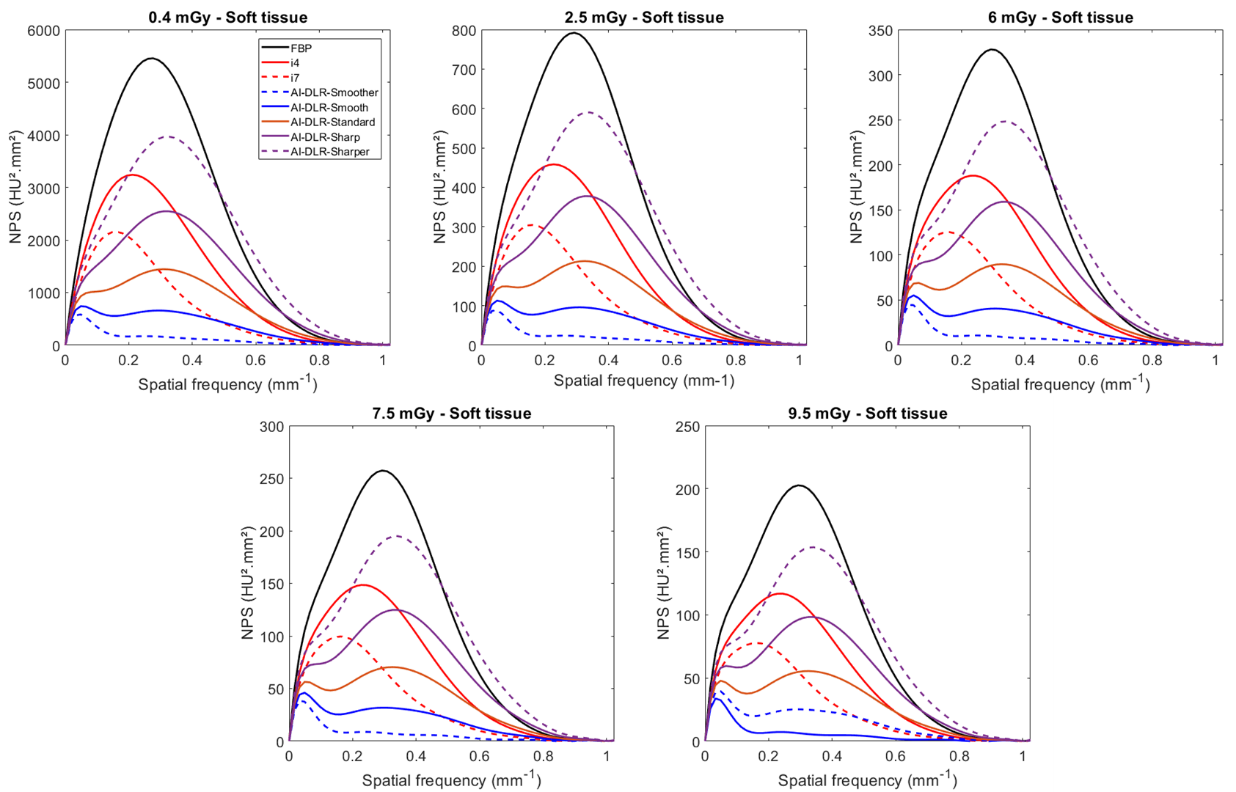


- *Lung kernel*


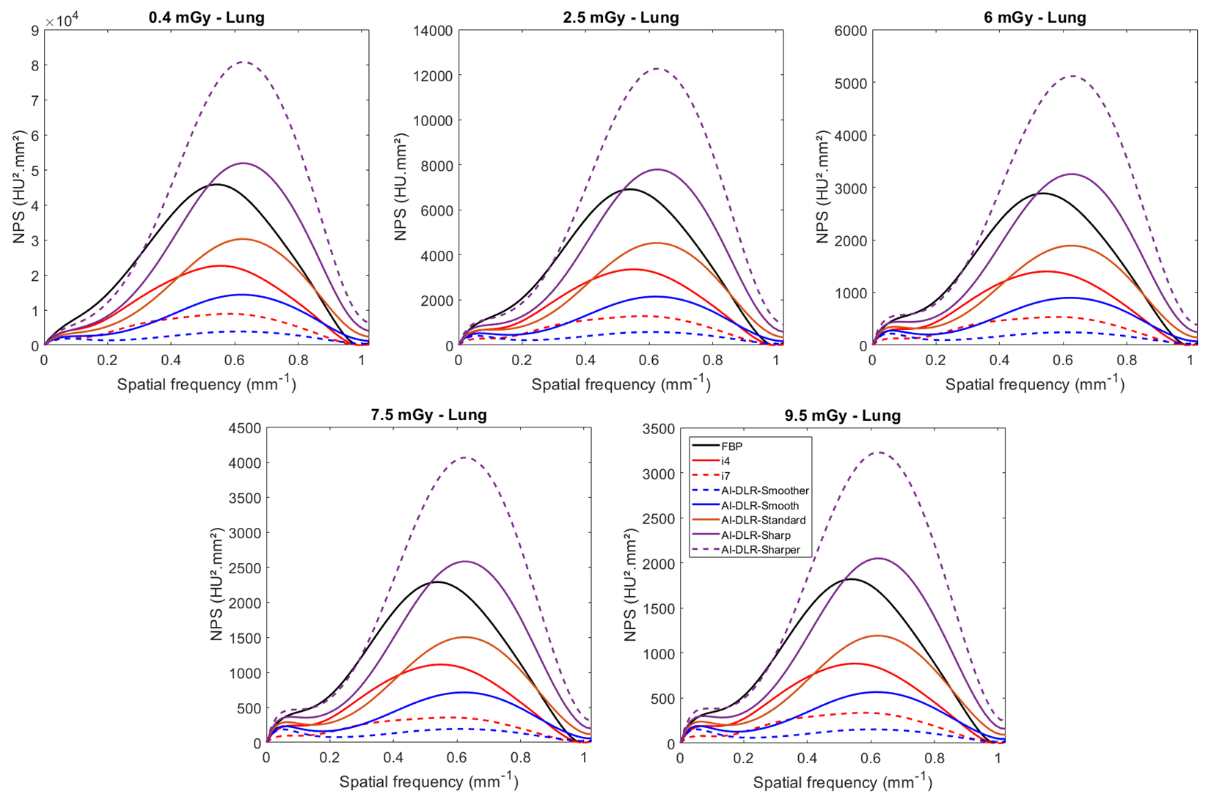


**Figure 2-SM.** Task-based transfer function (TTF) curves of the polyethylene and the air inserts obtained for all dose levels, all reconstruction types and both reconstruction kernels

- *Air insert*


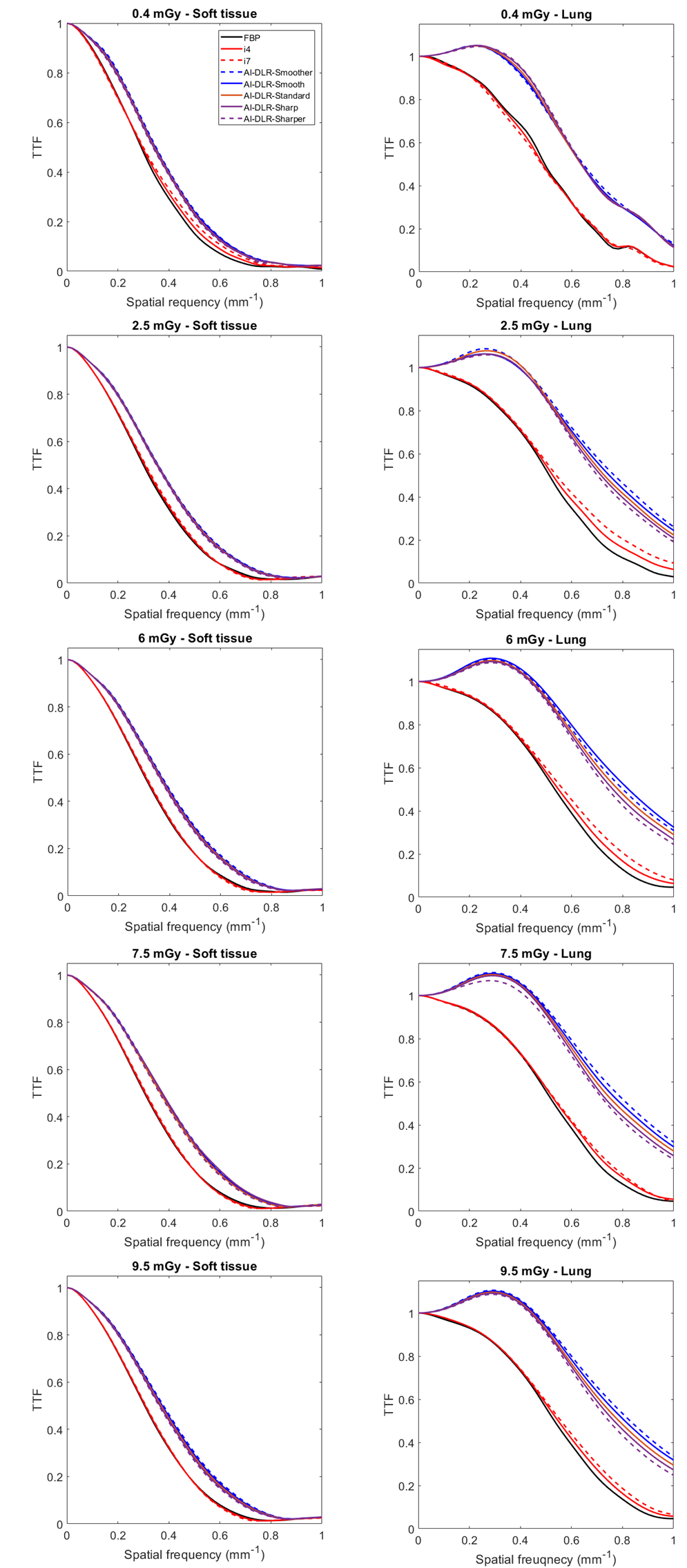


- *Polyethylene insert*


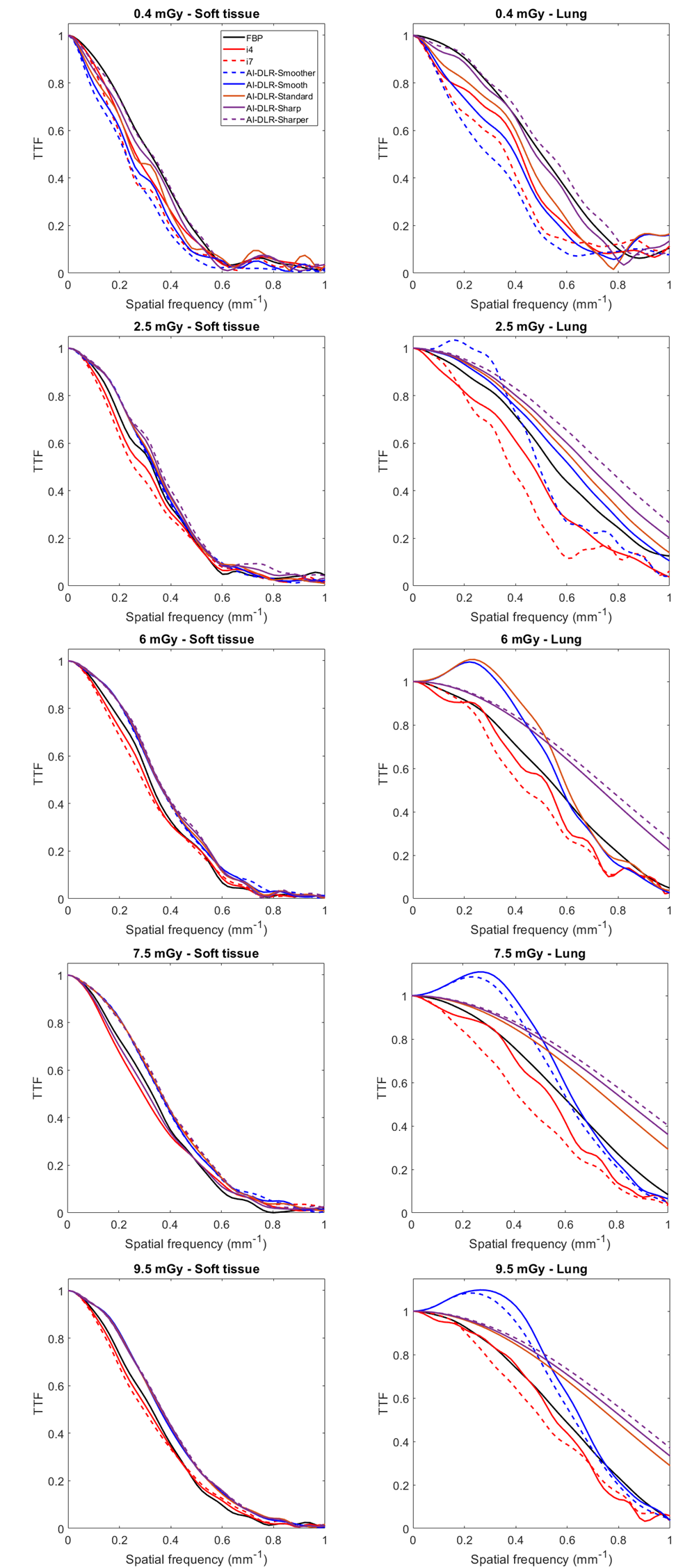

Supplement: Supplementary file 1 — Figure 1‐SM. Noise power spectrum (NPS) curves obtained for all dose levels, all reconstruction types and both reconstruction kernels Figure 2‐SM. Task‐based transfer function (TTF) curves of the polyethylene and the air inserts obtained for all dose levels, all reconstruction types and both reconstruction kernels [file MP-49-5052-s001.docx]
